# Supplementary material for: Prolonged Social Isolation, Started Early in Life, Impairs Cognitive Abilities in Rats Depending on Sex
Source: Brain Sci. 2020 Oct 30;10(11):799. doi: 10.3390/brainsci10110799 (PMC7692092; doi:10.3390/brainsci10110799)
Supplement: Supplementary file 1 [file brainsci-10-00799-s001.zip › Supplement updated final.docx]

**Figure S1.** The total distance traveled (cm) for 10 minutes in rats of all groups in the automated open field test. The horizontal axis indicates the age of the rats.

**Table S1.** The total distance traveled (cm) for 10 minutes and in the first 3 minutes in the automated open field (mean ± SEM). The results are shown in **Figure 2 (**sex × housing interaction) and **Figure S1** (all groups).

| **Age** | **Group** | **Duration** | **Distance traveled, cm** | **N** |
| --- | --- | --- | --- | --- |
| 1 month | Group-housed females | 10 minutes | 1813 ± 134 | 15 |
| 1 month | Group-housed females | 3 minutes | 942 ± 63 | 15 |
| 1 month | Group-housed males | 10 minutes | 1801 ± 156 | 20 |
| 1 month | Group-housed males | 3 minutes | 943 ± 57 | 20 |
| 1 month | Single-housed females | 10 minutes | 1657 ± 129 | 16 |
| 1 month | Single-housed females | 3 minutes | 863 ± 61 | 16 |
| 1 month | Single-housed males | 10 minutes | 1859 ± 129 | 16 |
| 1 month | Single-housed males | 3 minutes | 913 ± 61 | 16 |
| 3 months | Group-housed females | 10 minutes | 2855 ± 196 | 15 |
| 3 months | Group-housed females | 3 minutes | 1257 ± 76 | 15 |
| 3 months | Group-housed males | 10 minutes | 1723 ± 170 | 20 |
| 3 months | Group-housed males | 3 minutes | 811 ± 66 | 20 |
| 3 months | Single-housed females | 10 minutes | 2456 ± 190 | 16 |
| 3 months | Single-housed females | 3 minutes | 1021 ± 74 | 16 |
| 3 months | Single-housed males | 10 minutes | 2111 ± 190 | 16 |
| 3 months | Single-housed males | 3 minutes | 869 ± 74 | 16 |
| 5 months | Group-housed females | 10 minutes | 2666 ± 205 | 15 |
| 5 months | Group-housed females | 3 minutes | 1197 ± 80 | 15 |
| 5 months | Group-housed males | 10 minutes | 1469 ± 178 | 20 |
| 5 months | Group-housed males | 3 minutes | 671 ± 69 | 20 |
| 5 months | Single-housed females | 10 minutes | 2569 ± 199 | 16 |
| 5 months | Single-housed females | 3 minutes | 1111 ± 77 | 16 |
| 5 months | Single-housed males | 10 minutes | 1656 ± 199 | 16 |
| 5 months | Single-housed males | 3 minutes | 830 ± 77 | 16 |
| 8.5 months | Group-housed females | 10 minutes | 2180 ± 180 | 15 |
| 8.5 months | Group-housed females | 3 minutes | 987 ± 80 | 15 |
| 8.5 months | Group-housed males | 10 minutes | 1479 ± 156 | 20 |
| 8.5 months | Group-housed males | 3 minutes | 684 ± 69 | 20 |
| 8.5 months | Single-housed females | 10 minutes | 2517 ± 174 | 16 |
| 8.5 months | Single-housed females | 3 minutes | 1118 ± 77 | 16 |
| 8.5 months | Single-housed males | 10 minutes | 1570 ± 174 | 16 |
| 8.5 months | Single-housed males | 3 minutes | 765 ± 77 | 16 |

**Table S2.** The distance traveled (cm) by female and male rats for 10 minutes and in the first 3 minutes in the automated open field test (mean ± SEM). The results of *post hoc* analysis are shown in **Figure 3**.

| **Age** | **Group** | **Minute** | **Distance traveled, cm** | **N** |
| --- | --- | --- | --- | --- |
| 3 months | Females | 1 | 511 ± 22 | 33 |
| 3 months | Females | 2 | 339 ± 23 | 33 |
| 3 months | Females | 3 | 289 ± 23 | 33 |
| 3 months | Females | 4 | 284 ± 25 | 33 |
| 3 months | Females | 5 | 253 ± 22 | 33 |
| 3 months | Females | 6 | 257 ± 20 | 33 |
| 3 months | Females | 7 | 228 ± 21 | 33 |
| 3 months | Females | 8 | 184 ± 20 | 33 |
| 3 months | Females | 9 | 173 ± 24 | 33 |
| 3 months | Females | 10 | 137 ± 19 | 33 |
| 3 months | Males | 1 | 396 ± 21 | 36 |
| 3 months | Males | 2 | 265 ± 22 | 36 |
| 3 months | Males | 3 | 179 ± 22 | 36 |
| 3 months | Males | 4 | 169 ± 23 | 36 |
| 3 months | Males | 5 | 163 ± 21 | 36 |
| 3 months | Males | 6 | 174 ± 18 | 36 |
| 3 months | Males | 7 | 151 ± 19 | 36 |
| 3 months | Males | 8 | 149 ± 19 | 36 |
| 3 months | Males | 9 | 156 ± 22 | 36 |
| 3 months | Males | 10 | 116 ± 17 | 36 |
| 8.5 months | Females | 1 | 497 ± 25 | 33 |
| 8.5 months | Females | 2 | 314 ± 24 | 33 |
| 8.5 months | Females | 3 | 261 ± 19 | 33 |
| 8.5 months | Females | 4 | 237 ± 21 | 33 |
| 8.5 months | Females | 5 | 208 ± 23 | 33 |
| 8.5 months | Females | 6 | 194 ± 19 | 33 |
| 8.5 months | Females | 7 | 179 ± 21 | 33 |
| 8.5 months | Females | 8 | 190 ± 21 | 33 |
| 8.5 months | Females | 9 | 173 ± 17 | 33 |
| 8.5 months | Females | 10 | 155 ± 17 | 33 |
| 8.5 months | Males | 1 | 337 ± 24 | 36 |
| 8.5 months | Males | 2 | 189 ± 23 | 36 |
| 8.5 months | Males | 3 | 154 ± 19 | 36 |
| 8.5 months | Males | 4 | 106 ± 20 | 36 |
| 8.5 months | Males | 5 | 134 ± 22 | 36 |
| 8.5 months | Males | 6 | 139 ± 19 | 36 |
| 8.5 months | Males | 7 | 114 ± 20 | 36 |
| 8.5 months | Males | 8 | 122 ± 20 | 36 |
| 8.5 months | Males | 9 | 133 ± 17 | 36 |
| 8.5 months | Males | 10 | 97 ± 16 | 36 |

**Table S3.** The total number of rearing postures over 10 minutes and in the first 3 minutes in female and male rats in the automated open field test (mean ± SEM). The results of *post hoc* analysis are shown in **Figure 4**.

| **Age** | **Group** | **Duration** | **Number of rearing postures** | **N** |
| --- | --- | --- | --- | --- |
| 1 month | Females | 10 minutes | 52.6 ± 2.2 | 31 |
| 1 month | Females | 3 minutes | 21.8 ± 0.7 | 31 |
| 1 month | Males | 10 minutes | 59.1 ± 2.1 | 36 |
| 1 month | Males | 3 minutes | 24.1 ± 0.7 | 36 |
| 3 months | Females | 10 minutes | 41.3 ± 2.4 | 31 |
| 3 months | Females | 3 minutes | 17.4 ± 1.0 | 31 |
| 3 months | Males | 10 minutes | 35.9 ± 2.3 | 36 |
| 3 months | Males | 3 minutes | 14.7 ± 1.0 | 36 |
| 5 months | Females | 10 minutes | 41.3 ± 2.4 | 31 |
| 5 months | Females | 3 minutes | 17.6 ± 0.9 | 31 |
| 5 months | Males | 10 minutes | 30.8 ± 2.2 | 36 |
| 5 months | Males | 3 minutes | 13.5 ± 0.9 | 36 |
| 8.5 months | Females | 10 minutes | 41.3 ± 2.2 | 31 |
| 8.5 months | Females | 3 minutes | 17.6 ± 0.9 | 31 |
| 8.5 months | Males | 10 minutes | 33.8 ± 2.0 | 36 |
| 8.5 months | Males | 3 minutes | 14.1 ± 0.9 | 36 |

**Table S4.** The total distance traveled (number of squares) for 3 minutes in rats in the classic open field test (mean ± SEM): sex and housing factors. The results of *post hoc* analysis are shown in **Figure 5**.

| **Age** | **Group** | **Distance traveled, number of squares** | **N** |
| --- | --- | --- | --- |
| 1 month | Females | 63.9 ± 4.1 | 32 |
| 1 month | Males | 53.7 ± 3.9 | 36 |
| 3 months | Females | 56.1 ± 3.5 | 32 |
| 3 months | Males | 34.0 ± 3.3 | 36 |
| 5 months | Females | 47.9 ± 3.3 | 32 |
| 5 months | Males | 24.8 ± 3.1 | 36 |
| 8.5 months | Females | 42.2 ± 3.2 | 32 |
| 8.5 months | Males | 25.2 ± 3.1 | 36 |
| 1 month | Group-housed | 56.2 ± 3.9 | 36 |
| 1 month | Single-housed | 61.3 ± 4.1 | 32 |
| 3 months | Group-housed | 39.2 ± 3.3 | 36 |
| 3 months | Single-housed | 50.8 ± 3.5 | 32 |
| 5 months | Group-housed | 33.1 ± 3.1 | 36 |
| 5 months | Single-housed | 39.6 ± 3.3 | 32 |
| 8.5 months | Group-housed | 29.7 ± 3.1 | 36 |
| 8.5 months | Single-housed | 37.8 ± 3.2 | 32 |

**Figure S2.** The total distance traveled (number of squares) for 3 minutes in rats of all groups in the classic open field test. The horizontal axis indicates the age of the rats.

**Table S4a.** The total distance traveled (number of squares) for 3 minutes in rats of all groups in the classic open field test (mean ± SEM). The results are shown in **Fig. S2**.

| **Age** | **Group** | **Distance traveled, number of squares** | **N** |
| --- | --- | --- | --- |
| 1 month | Group-housed females | 63.9 ± 5.9 | 16 |
| 1 month | Group-housed males | 63.9 ± 5.0 | 16 |
| 1 month | Single-housed females | 48.6 ± 4.7 | 20 |
| 1 month | Single-housed males | 58.8 ± 4.6 | 16 |
| 3 months | Group-housed females | 48.2 ± 5.9 | 16 |
| 3 months | Group-housed males | 63.9 ± 5.0 | 16 |
| 3 months | Single-housed females | 30.3 ± 4.7 | 20 |
| 3 months | Single-housed males | 37.7 ± 4.6 | 16 |
| 5 months | Group-housed females | 44.0 ± 5.2 | 16 |
| 5 months | Group-housed males | 51.8 ± 4.4 | 16 |
| 5 months | Single-housed females | 22.2 ± 4.2 | 20 |
| 5 months | Single-housed males | 27.4 ± 4.1 | 16 |
| 8.5 months | Group-housed females | 40.6 ± 5.9 | 16 |
| 8.5 months | Group-housed males | 43.9 ± 5.0 | 16 |
| 8.5 months | Single-housed females | 18.8 ± 4.7 | 20 |
| 8.5 months | Single-housed males | 31.7 ± 4.6 | 16 |

**Table S5.** Reactivity to novelty (the ratio of the distance traveled in the first minute to the distance traveled in the fourth minute in the classic open field test) (mean ± SEM). The results of *post hoc* analysis are shown in **Figure 6**.

| **Age** | **Group** | **Reactivity to novelty** | **N** |
| --- | --- | --- | --- |
| 1 month | Group-housed | 1.0 ± 0.1 | 34 |
| 1 month | Single-housed | 0.8 ± 0.1 | 31 |
| 3 months | Group-housed | 1.5 ± 0.1 | 34 |
| 3 months | Single-housed | 1.2 ± 0.2 | 31 |
| 5 months | Group-housed | 2.3 ± 0.3 | 34 |
| 5 months | Single-housed | 1.1 ± 0.3 | 31 |
| 8.5 months | Group-housed | 2.0 ± 0.2 | 34 |
| 8.5 months | Single-housed | 1.2 ± 0.2 | 31 |

**Table S6.** Overall distance traveled in the Morris water maze (MWM) (mean ± SEM); 5.5-month-old rats: Training day 1–4 and Probe 1; 9.5-month-old rats: Probe 2, the Reminder trials, and Probe 3. The results of *post hoc* analysis are shown in **Figure 7A**.

| **Stage** | **Group** | **Distance traveled, cm** | **N** |
| --- | --- | --- | --- |
| Training day 1 | Group-housed females | 1001 ± 83 | 17 |
| Training day 1 | Group-housed males | 1135 ± 102 | 20 |
| Training day 1 | Single-housed females | 709 ± 159 | 16 |
| Training day 1 | Single-housed males | 1195 ± 58 | 16 |
| Training day 2 | Group-housed females | 661 ± 101 | 17 |
| Training day 2 | Group-housed males | 655 ± 76 | 20 |
| Training day 2 | Single-housed females | 752 ± 91 | 16 |
| Training day 2 | Single-housed males | 660 ± 73 | 16 |
| Training day 3 | Group-housed females | 532 ± 58 | 17 |
| Training day 3 | Group-housed males | 325 ± 38 | 20 |
| Training day 3 | Single-housed females | 448 ± 60 | 16 |
| Training day 3 | Single-housed males | 301 ± 23 | 16 |
| Training day 4 | Group-housed females | 382 ± 66 | 17 |
| Training day 4 | Group-housed males | 368 ± 43 | 20 |
| Training day 4 | Single-housed females | 315 ± 46 | 16 |
| Training day 4 | Single-housed males | 290 ± 30 | 16 |
| Probe 1 | Group-housed females | 3072 ± 115 | 17 |
| Probe 1 | Group-housed males | 2882 ± 77 | 20 |
| Probe 1 | Single-housed females | 2839 ± 139 | 16 |
| Probe 1 | Single-housed males | 2904 ± 89 | 16 |
| Probe 2 | Group-housed females | 2780 ± 132 | 17 |
| Probe 2 | Group-housed males | 2654 ± 64 | 20 |
| Probe 2 | Single-housed females | 2612 ± 122 | 16 |
| Probe 2 | Single-housed males | 2597 ± 99 | 16 |
| Reminder trial | Group-housed females | 699 ± 97 | 17 |
| Reminder trial | Group-housed males | 811 ± 132 | 20 |
| Reminder trial | Single-housed females | 805 ± 108 | 16 |
| Reminder trial | Single-housed males | 639 ± 121 | 16 |
| Probe 3 | Group-housed females | 2956 ± 111 | 17 |
| Probe 3 | Group-housed males | 2640 ± 90 | 20 |
| Probe 3 | Single-housed females | 2846 ± 136 | 16 |
| Probe 3 | Single-housed males | 2722 ± 121 | 16 |

**Table S7.** Average speed in the MWM (mean ± SEM); 5.5-month-old rats: Training day 1–4 and Probe 1; 9.5-month-old rats: Probe 2, the Reminder trials, and Probe 3. The results of *post hoc* analysis are shown in **Figure 7B**.

| **Stage** | **Group** | **Average speed, cm/sec** | **N** |
| --- | --- | --- | --- |
| Training day 1 | Group-housed females | 21.6 ± 0.8 | 17 |
| Training day 1 | Group-housed males | 18.5 ± 0.7 | 20 |
| Training day 1 | Single-housed females | 9.9 ± 1.7 | 16 |
| Training day 1 | Single-housed males | 19.7 ± 0.6 | 16 |
| Training day 2 | Group-housed females | 23.4 ± 1.0 | 17 |
| Training day 2 | Group-housed males | 22.5 ± 0.9 | 20 |
| Training day 2 | Single-housed females | 23.2 ± 1.1 | 16 |
| Training day 2 | Single-housed males | 22.7 ± 1.1 | 16 |
| Training day 3 | Group-housed females | 24.9 ± 1.2 | 17 |
| Training day 3 | Group-housed males | 22.6 ± 1.0 | 20 |
| Training day 3 | Single-housed females | 22.5 ± 0.7 | 16 |
| Training day 3 | Single-housed males | 22.6 ± 1.3 | 16 |
| Training day 4 | Group-housed females | 24.6 ± 1.3 | 17 |
| Training day 4 | Group-housed males | 23.3 ± 1.2 | 20 |
| Training day 4 | Single-housed females | 23.2 ± 1.2 | 16 |
| Training day 4 | Single-housed males | 22.4 ± 1.1 | 16 |
| Probe 1 | Group-housed females | 25.6 ± 1.0 | 17 |
| Probe 1 | Group-housed males | 24.0 ± 0.6 | 20 |
| Probe 1 | Single-housed females | 23.7 ± 1.2 | 16 |
| Probe 1 | Single-housed males | 24.2 ± 0.7 | 16 |
| Probe 2 | Group-housed females | 23.2 ± 1.1 | 17 |
| Probe 2 | Group-housed males | 22.1 ± 0.5 | 20 |
| Probe 2 | Single-housed females | 21.8 ± 1.0 | 16 |
| Probe 2 | Single-housed males | 21.6 ± 0.8 | 16 |
| Reminder trial | Group-housed females | 25.9 ± 1.3 | 17 |
| Reminder trial | Group-housed males | 27.2 ± 1.2 | 20 |
| Reminder trial | Single-housed females | 23.1 ± 1.2 | 16 |
| Reminder trial | Single-housed males | 23.7 ± 1.2 | 16 |
| Probe 3 | Group-housed females | 24.6 ± 0.9 | 17 |
| Probe 3 | Group-housed males | 22.0 ± 0.8 | 20 |
| Probe 3 | Single-housed females | 23.7 ± 1.1 | 16 |
| Probe 3 | Single-housed males | 22.7 ± 1.0 | 16 |

**Table S8.** The latency to reach the area around the platform site (mean ± SEM); 5.5-month-old rats: Training day 1–4 and Probe 1; 9.5-month-old rats: Probe 2, the Reminder trials, and Probe 3. The results of *post hoc* analysis are shown in **Figure 7C**.

| **Stage** | **Group** | **Latency to reach the area around the platform site, sec** | **N** |
| --- | --- | --- | --- |
| Training day 1 | Group-housed females | 24.4 ± 2.6 | 17 |
| Training day 1 | Group-housed males | 31.8 ± 2.7 | 20 |
| Training day 1 | Single-housed females | 40.8 ± 4.0 | 16 |
| Training day 1 | Single-housed males | 36.1 ± 3.2 | 16 |
| Training day 2 | Group-housed females | 15.1 ± 2.1 | 17 |
| Training day 2 | Group-housed males | 16.5 ± 2.3 | 20 |
| Training day 2 | Single-housed females | 17.5 ± 2.3 | 16 |
| Training day 2 | Single-housed males | 16.6 ± 1.6 | 16 |
| Training day 3 | Group-housed females | 10.0 ± 1.1 | 17 |
| Training day 3 | Group-housed males | 10.1 ± 1.0 | 20 |
| Training day 3 | Single-housed females | 10.1 ± 1.3 | 16 |
| Training day 3 | Single-housed males | 9.3 ± 0.9 | 16 |
| Training day 4 | Group-housed females | 9.8 ± 1.1 | 17 |
| Training day 4 | Group-housed males | 11.3 ± 1.4 | 20 |
| Training day 4 | Single-housed females | 9.3 ± 1.1 | 16 |
| Training day 4 | Single-housed males | 9.2 ± 1.0 | 16 |
| Probe 1 | Group-housed females | 14.6 ± 3.2 | 17 |
| Probe 1 | Group-housed males | 11.1 ± 2.0 | 20 |
| Probe 1 | Single-housed females | 8.5 ± 1.1 | 16 |
| Probe 1 | Single-housed males | 13.2 ± 2.8 | 16 |
| Probe 2 | Group-housed females | 24.8 ± 6.8 | 17 |
| Probe 2 | Group-housed males | 24.0 ± 4.9 | 20 |
| Probe 2 | Single-housed females | 27.6 ± 4.4 | 16 |
| Probe 2 | Single-housed males | 20.4 ± 3.4 | 16 |
| Reminder trial | Group-housed females | 16.2 ± 2.1 | 17 |
| Reminder trial | Group-housed males | 12.5 ± 2.6 | 20 |
| Reminder trial | Single-housed females | 23.9 ± 4.5 | 16 |
| Reminder trial | Single-housed males | 12.5 ± 1.4 | 16 |
| Probe 3 | Group-housed females | 8.9 ± 1.6 | 17 |
| Probe 3 | Group-housed males | 10.3 ± 1.7 | 20 |
| Probe 3 | Single-housed females | 21.5 ± 5.9 | 16 |
| Probe 3 | Single-housed males | 10.5 ± 1.5 | 16 |

**Table S9.** The latency to reach the platform site (mean ± SEM); 5.5-month-old rats: Training day 1–4 and Probe 1; 9.5-month-old rats: Probe 2, the Reminder trials, and Probe 3. The results of *post hoc* analysis are shown in **Figure 7D**.

| **Stage** | **Group** | **Latency to reach the platform site, sec** | **N** |
| --- | --- | --- | --- |
| Training day 1 | Group-housed females | 26.9 ± 2.6 | 17 |
| Training day 1 | Group-housed males | 38.6 ± 3.1 | 19 |
| Training day 1 | Single-housed females | 41.7 ± 5.3 | 16 |
| Training day 1 | Single-housed males | 42.8 ± 3.6 | 16 |
| Training day 2 | Group-housed females | 24.7 ± 2.9 | 17 |
| Training day 2 | Group-housed males | 28.0 ± 3.1 | 20 |
| Training day 2 | Single-housed females | 26.2 ± 1.8 | 16 |
| Training day 2 | Single-housed males | 24.6 ± 2.9 | 16 |
| Training day 3 | Group-housed females | 19.9 ± 2.1 | 17 |
| Training day 3 | Group-housed males | 14.3 ± 1.5 | 20 |
| Training day 3 | Single-housed females | 17.7 ± 1.9 | 16 |
| Training day 3 | Single-housed males | 13.7 ± 1.3 | 16 |
| Training day 4 | Group-housed females | 14.5 ± 2.2 | 17 |
| Training day 4 | Group-housed males | 15.9 ± 1.9 | 20 |
| Training day 4 | Single-housed females | 12.8 ± 1.5 | 16 |
| Training day 4 | Single-housed males | 13.4 ± 1.4 | 16 |
| Probe 1 | Group-housed females | 24.6 ± 4.5 | 16 |
| Probe 1 | Group-housed males | 20.6 ± 4.2 | 19 |
| Probe 1 | Single-housed females | 17.8 ± 4.9 | 16 |
| Probe 1 | Single-housed males | 29.2 ± 7.9 | 16 |
| Probe 2 | Group-housed females | 28.2 ± 10.6 | 12 |
| Probe 2 | Group-housed males | 40.5 ± 7.0 | 17 |
| Probe 2 | Single-housed females | 42.2 ± 6.9 | 14 |
| Probe 2 | Single-housed males | 27.9 ± 5.6 | 13 |
| Reminder trial | Group-housed females | 27.3 ± 3.7 | 17 |
| Reminder trial | Group-housed males | 27.3 ± 4.9 | 20 |
| Reminder trial | Single-housed females | 30.1 ± 4.6 | 16 |
| Reminder trial | Single-housed males | 23.1 ± 4.1 | 16 |
| Probe 3 | Group-housed females | 23.4 ± 5.7 | 16 |
| Probe 3 | Group-housed males | 25.9 ± 7.0 | 20 |
| Probe 3 | Single-housed females | 23.3 ± 5.6 | 15 |
| Probe 3 | Single-housed males | 26.8 ± 5.6 | 15 |

**Table S10.** Relative time spent in the target quadrant; 5.5-month-old rats: Training day 1–4 and Probe 1; 9.5-month-old rats: Probe 2, the Reminder trials, and Probe 3. The results of *post hoc* analysis are shown in **Figure 7E**.

| **Stage** | **Group** | **Relative time spent in the target quadrant, %** | **N** |
| --- | --- | --- | --- |
| Training day 1 | Group-housed females | 37.3 ± 2.0 | 17 |
| Training day 1 | Group-housed males | 34.2 ± 2.3 | 20 |
| Training day 1 | Single-housed females | 33.7 ± 2.8 | 16 |
| Training day 1 | Single-housed males | 36.2 ± 2.4 | 16 |
| Training day 2 | Group-housed females | 39.0 ± 2.0 | 17 |
| Training day 2 | Group-housed males | 36.5 ± 2.8 | 20 |
| Training day 2 | Single-housed females | 39.4 ± 2.8 | 16 |
| Training day 2 | Single-housed males | 39.5 ± 3.0 | 16 |
| Training day 3 | Group-housed females | 41.3 ± 2.3 | 17 |
| Training day 3 | Group-housed males | 45.7 ± 2.2 | 20 |
| Training day 3 | Single-housed females | 49.9 ± 2.4 | 16 |
| Training day 3 | Single-housed males | 43.4 ± 2.7 | 16 |
| Training day 4 | Group-housed females | 46.3 ± 1.8 | 17 |
| Training day 4 | Group-housed males | 44.3 ± 2.9 | 20 |
| Training day 4 | Single-housed females | 47.6 ± 2.8 | 16 |
| Training day 4 | Single-housed males | 50.2 ± 2.5 | 16 |
| Probe 1 | Group-housed females | 34.3 ± 1.6 | 17 |
| Probe 1 | Group-housed males | 36.1 ± 1.4 | 20 |
| Probe 1 | Single-housed females | 43.3 ± 2.4 | 16 |
| Probe 1 | Single-housed males | 37.6 ± 2.0 | 16 |
| Probe 2 | Group-housed females | 21.1 ± 1.5 | 17 |
| Probe 2 | Group-housed males | 22.8 ± 1.3 | 20 |
| Probe 2 | Single-housed females | 25.2 ± 2.1 | 16 |
| Probe 2 | Single-housed males | 22.8 ± 1.5 | 16 |
| Reminder trial | Group-housed females | 29.0 ± 2.1 | 17 |
| Reminder trial | Group-housed males | 31.8 ± 1.9 | 20 |
| Reminder trial | Single-housed females | 32.2 ± 1.5 | 16 |
| Reminder trial | Single-housed males | 31.0 ± 3.0 | 16 |
| Probe 3 | Group-housed females | 28.9 ± 2.4 | 17 |
| Probe 3 | Group-housed males | 29.8 ± 2.2 | 20 |
| Probe 3 | Single-housed females | 27.5 ± 2.3 | 16 |
| Probe 3 | Single-housed males | 30.1 ± 2.1 | 16 |

**Table S11.** Relative time spent in the border area; 5.5-month-old rats: Training day 1–4 and Probe 1; 9.5-month-old rats: Probe 2, the Reminder trials, and Probe 3. The results of *post hoc* analysis are shown in **Figure 7F**.

| **Stage** | **Group** | **Relative time spent in the border area, %** | **N** |
| --- | --- | --- | --- |
| Training day 1 | Group-housed females | 56.1 ± 2.3 | 17 |
| Training day 1 | Group-housed males | 56.5 ± 2.5 | 20 |
| Training day 1 | Single-housed females | 66.9 ± 3.6 | 16 |
| Training day 1 | Single-housed males | 63.0 ± 2.1 | 16 |
| Training day 2 | Group-housed females | 38.6 ± 3.0 | 17 |
| Training day 2 | Group-housed males | 35.9 ± 3.6 | 20 |
| Training day 2 | Single-housed females | 48.5 ± 3.4 | 16 |
| Training day 2 | Single-housed males | 37.5 ± 4.0 | 16 |
| Training day 3 | Group-housed females | 38.9 ± 3.9 | 17 |
| Training day 3 | Group-housed males | 34.1 ± 3.9 | 20 |
| Training day 3 | Single-housed females | 45.0 ± 4.0 | 16 |
| Training day 3 | Single-housed males | 38.5 ± 4.1 | 16 |
| Training day 4 | Group-housed females | 37.1 ± 3.3 | 17 |
| Training day 4 | Group-housed males | 34.4 ± 3.5 | 20 |
| Training day 4 | Single-housed females | 41.7 ± 3.4 | 16 |
| Training day 4 | Single-housed males | 45.6 ± 4.2 | 16 |
| Probe 1 | Group-housed females | 39.8 ± 3.1 | 17 |
| Probe 1 | Group-housed males | 35.9 ± 2.9 | 20 |
| Probe 1 | Single-housed females | 41.6 ± 2.8 | 16 |
| Probe 1 | Single-housed males | 36.7 ± 3.2 | 16 |
| Probe 2 | Group-housed females | 36.4 ± 2.1 | 17 |
| Probe 2 | Group-housed males | 36.5 ± 2.8 | 20 |
| Probe 2 | Single-housed females | 41.5 ± 4.8 | 16 |
| Probe 2 | Single-housed males | 35.2 ± 3.3 | 16 |
| Reminder trial | Group-housed females | 31.9 ± 4.5 | 17 |
| Reminder trial | Group-housed males | 27.6 ± 3.5 | 20 |
| Reminder trial | Single-housed females | 38.5 ± 5.6 | 16 |
| Reminder trial | Single-housed males | 25.4 ± 4.2 | 16 |
| Probe 3 | Group-housed females | 30.7 ± 2.9 | 17 |
| Probe 3 | Group-housed males | 28.0 ± 3.0 | 20 |
| Probe 3 | Single-housed females | 40.4 ± 5.3 | 16 |
| Probe 3 | Single-housed males | 24.6 ± 2.8 | 16 |

**Table S12.** The latency to enter the dark compartment of the arena in the passive avoidance paradigm (mean ± SEM). The results of *post hoc* analysis are shown in **Figure 8**.

| **Stage** | **Group** | **Latency to enter the dark compartment** | **N** |
| --- | --- | --- | --- |
| Habituation | Group-housed | 38.5 ± 4.4 | 27 |
| Habituation | Single-housed | 23.0 ± 4.8 | 32 |
| Acquisition | Group-housed | 16.1 ± 2.0 | 27 |
| Acquisition | Single-housed | 13.6 ± 2.1 | 32 |
| Retention 1 | Group-housed | 137.9 ± 17.3 | 27 |
| Retention 1 | Single-housed | 29.8 ± 18.8 | 32 |
| Retention 2 | Group-housed | 124.4 ± 15.3 | 27 |
| Retention 2 | Single-housed | 19.8 ± 16.6 | 32 |
