# Supplementary figures and images for: Prolonged Social Isolation, Started Early in Life, Impairs Cognitive Abilities in Rats Depending on Sex
Source: Brain Sci. 2020 Oct 30;10(11):799. doi: 10.3390/brainsci10110799 (PMC7692092; doi:10.3390/brainsci10110799)

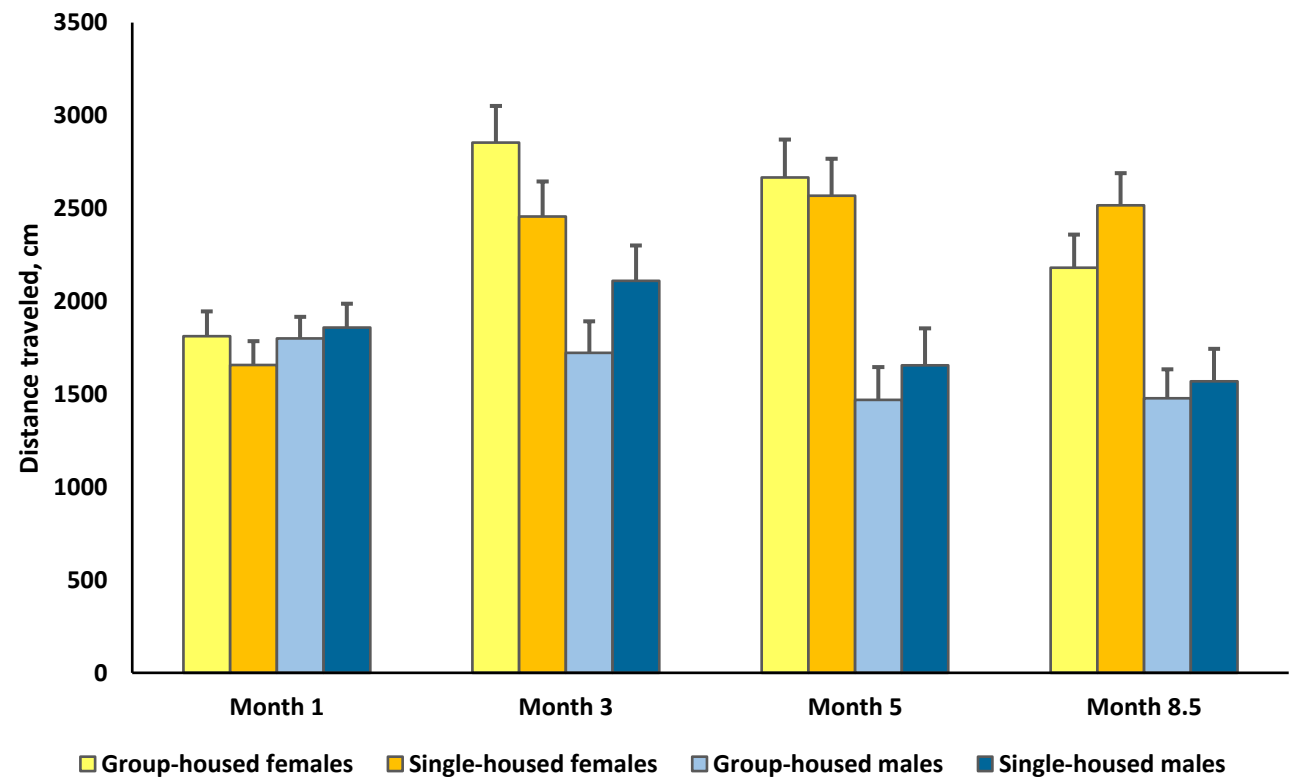

Supplement: Supplementary file 1 [file brainsci-10-00799-s001.zip › Figure S1.pdf]

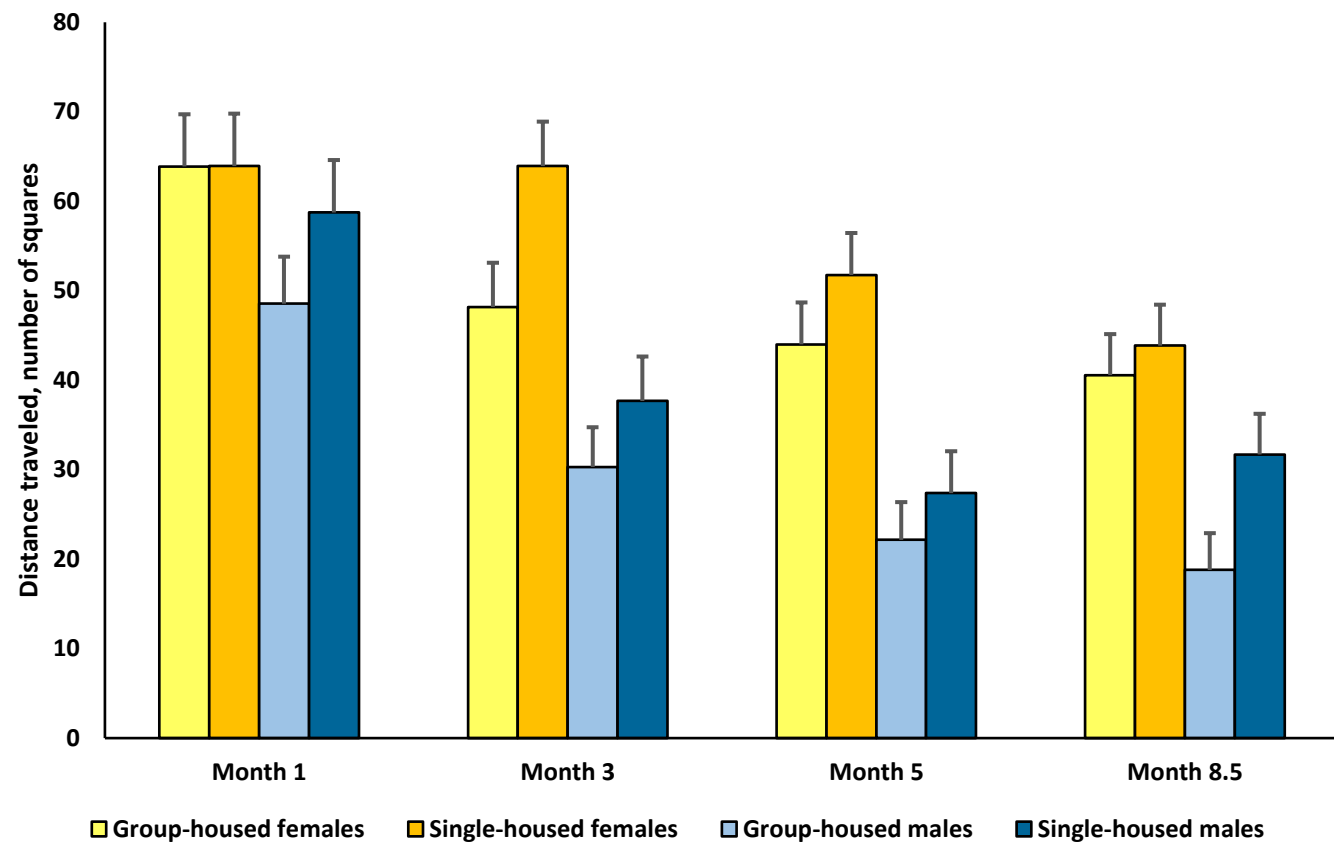

Supplement: Supplementary file 1 [file brainsci-10-00799-s001.zip › Figure S2.pdf]
